# Draft Genome Assembly of Two *Pseudoclavibacter helvolus* Strains, G8 and W3, Isolated from Slaughterhouse Environments

**DOI:** 10.1128/genomeA.00077-16

**Published:** 2016-03-31

**Authors:** Prem K. Raghupathi, Jakob Herschend, Henriette L. Røder, Søren J. Sørensen, Mette Burmølle

**Affiliations:** Section for Microbiology, Department of Biology, University of Copenhagen, Copenhagen, Denmark

## Abstract

We report the draft genome sequences of two *Pseudoclavibacter helvolus* strains. Strain G8 was isolated from a meat chopper and strain W3 isolated from the wall of a small slaughterhouse in Denmark. The two annotated genomes are 3.91 Mb and 4.00 Mb in size, respectively.

## GENOME ANNOUNCEMENT

*Pseudoclavibacter helvolus* is an irregular rod-shaped, Gram-positive, strictly aerobic and nonspore forming bacterium belonging to the family of *Microbacteriaceae* (1). The type strain *P. helvolus* DSM 20419 was originally isolated from butter (1). Here, we present the draft assembly of two *P. helvolus* strains G8 and W3, both isolated from a slaughterhouse in Denmark (2). The whole-genome sequencing libraries were prepared using the Nextera XT kit (Illumina, USA), according to the manufacturer’s recommendations, followed by sequencing as a part of the flowcell, as 2 × 250-base paired-end reads, using Illumina MiSeq (Illumina, USA) technology. The reads were cleaned and trimmed using CLC Genomics Workbench 7 (CLC bio, Denmark). Quality filtered reads were assembled using SPAdes v3.5.0 (3). The annotations on the resulting contigs were performed on the RAST server (4) and RNAmmer 1.2 (5) to check and screen for noncoding RNAs.

The genome assembly of *P. helvolus* G8 and W3 resulted in 125 and 195 contigs with 80× and 179× fold coverage, respectively. The average G+C content is 65% for both assemblies. The annotated results from G8 predicted 3,741 coding sequences with an average length of 898 bp (1,397 coding sequences [CDSs] have functional predictions), 13 tRNA-encoding genes, and 3 rRNA-encoding genes. The predictions from W3 included 3,836 coding sequences with an average length of 889 bp (1,373 CDSs have functional predictions), 13 tRNA-encoding genes, and 3 rRNA-encoding genes. Both strains G8 and W3 had single predicted copies of 16s, 23s, and 5s rRNA genes. RAST revealed the closest neighbors of both *P. helvolus* strains to be the marine *Actinobacterium* PHSC20C1, followed by *Sanguibacter keddieii* DSM 10542*.* Sequence based comparison showed >99.5% identity to each other and only ~<60% similarity to marine *Actinobacterium* PHSC20C1. There are 378 and 373 predicted subsystems in the genomes of G8 and W3, respectively. Metabolic network comparisons revealed 1,640 putative protein encoding genes (PEGs) conserved in both of the G8 and W3 genomes, out of which 23 PEGs unique to G8 and 25 PEGs unique to W3 were predicted. Both strains included genes belonging to various subsystems like amino acid and derivatives; clustering; fatty acid, lipids, and isoprenoids; carbohydrates; and stress responses, virulence, and diseases in their genome. Interestingly, strain G8 had higher PEGs for amino acid derivative mechanisms compared to strain W3. The finding that both of these genomes of *P. helvolus* have predicted sequences for clustered regularly interspaced short palindromic repeat (CRISPR) elements indicates a prominent influence of phage exposure being present in more active and densely populated animal gut environments. Further work with these genomes is expected to facilitate the identification and understanding of genes associated with adaptive mechanisms and biofilm formation of *P. helvolus* strains.

### Nucleotide sequence accession numbers.

The whole-genome shotgun projects for *P. helvolus* strains G8 and W3 have been deposited in the European Nucleotide Archive (ENA) under the contig accession numbers CZJY01000001 to CZJY01000125 and CZJS01000001 to CZJS01000195, respectively. The versions described in this paper are the first versions.
